# Statistical analysis of interatomic transfer integrals for exploring high-mobility organic semiconductors

**DOI:** 10.1080/14686996.2024.2354652

**Published:** 2024-05-17

**Authors:** Koki Ozawa, Tomoharu Okada, Hiroyuki Matsui

**Affiliations:** Research Center for Organic Electronics (ROEL), Yamagata University, Yonezawa, Japan

**Keywords:** Organic semiconductors, transfer integrals, density functional theory, materials informatics, organic transistors

## Abstract

Charge transport in organic semiconductors occurs via overlapping molecular orbitals quantified by transfer integrals. However, no statistical study of transfer integrals for a wide variety of molecules has been reported. Here we present a statistical analysis of transfer integrals for more than 27,000 organic compounds in the Cambridge Structural Database. Interatomic transfer integrals were used to identify substructures with high transfer integrals. As a result, thione and amine groups as in thiourea were found to exhibit high transfer integrals. Such compounds are considered as potential non-aromatic, water-soluble organic semiconductors.

## IMPACT STATEMENT

The analysis of interatomic transfer integrals for 27,718 organic compounds revealed that thione (S=R)–amine (NR_3_) and thione–thione interactions tend to increase transfer integrals and are suitable to high–mobility organic semiconductors.

## GRAPHICAL ABSTRACT

**Figure:**
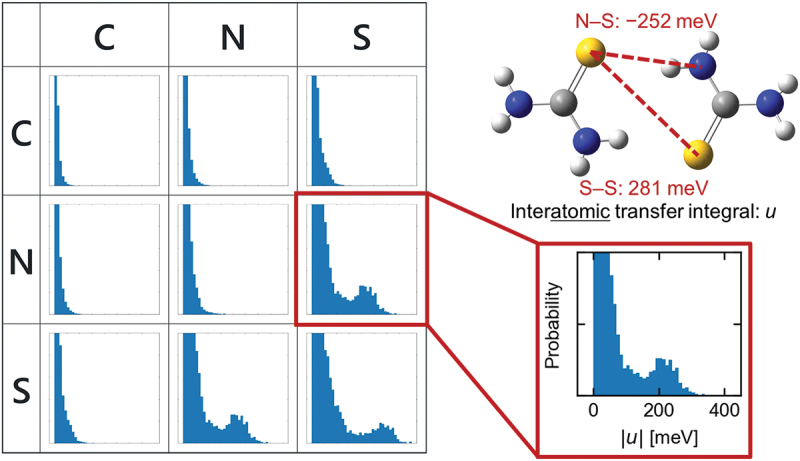

## Introduction

Organic semiconductors (OSCs) are suitable for large-area, flexible, and low-cost devices such as organic field-effect transistors (OFETs) [1,2]. Charge carrier mobility is the most important performance parameter of OSCs for OFETs because its value limits the range of applications [3–5]. Several OSCs have been reported to exhibit charge carrier mobilities >10 cm^2^ V^−1^ s^−1^ [6–10]. Transfer integrals (TIs), a parameter associated with charge carrier mobility, indicate the magnitude of electronic interaction between the orbitals of adjacent molecules and are roughly proportional to the overlap of orbitals [11]. Mobility (μ) is proportional to the square of the TI (*t*), $\mu \propto t^{2}$, based on the Einstein relation and semiclassical Marcus theory [12]. Therefore, increasing the TI value is a straightforward strategy for developing OSCs with high charge carrier mobility. However, the calculation of the TI value has been limited. Although the TIs of common OSCs such as pentacene and [1]benzothieno[3,2-*b*][1]benzothiophene (BTBT) have been reported [7,9,10,13–16], no statistical analysis of TIs for a large number of compounds is available for three reasons: (i) the calculation of TIs requires crystal structures, (ii) the computational cost is higher for dimers than those for monomers, (iii) methods to correlate between TIs and molecular structures are unknown. Recently, the growth of crystal structure databases and increasing computer performance have made exhaustive calculations possible [15,17–21].

Here we show the statistical analysis of TIs for 27,718 small molecules. The compounds were selected from the Cambridge Structural Database (CSD) [22] via prescreening adapted for p-type OSCs. Interatomic TIs were introduced to analyze the contribution of each atom to the intermolecular TI. The histograms of the interatomic TIs for the combinations of atomic elements show that sulfur – sulfur and sulfur – nitrogen pairs tend to have high TIs. Thione (S=R) and amine (NR_3_) structures were suitable for increasing TIs. Thiourea, for instance, has charge mobility of 0.2 cm^2^ V^−1^ s^−1^ [23,24].

## Methods

The CSD comprising X-ray and neutron diffraction analyses of >1.2 million crystal structures of organic compounds was used as a data source [22]. The primary screening was performed using ConQuest [25] and CSD Python API [22] to reduce the total calculation time following the mentioned conditions: (i) R factor <10%, (ii) single component, (iii) not polymers, (iv) possess 3D coordinates, (v) not disordered, (vi) not ionic, (vii) not powder structure, (viii) one or more bonds apart from a single bond, (ix) one or more rings, and (x) no heavy metal. Thus, ~220,000 molecules were extracted from CSD.

**Figure 1.**
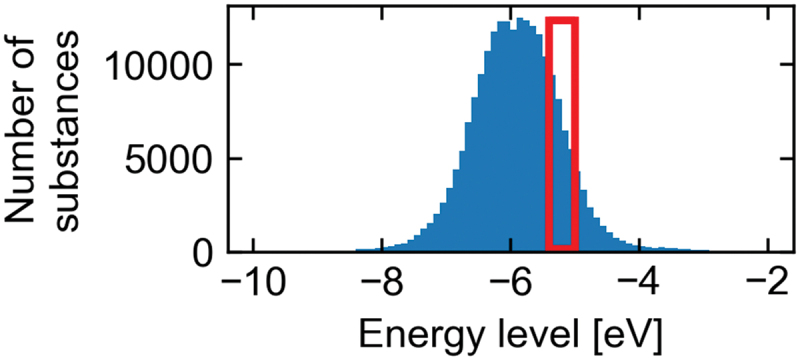
The HOMO levels of 220,000 compounds after primary screening.

Secondary screening was performed based on the highest occupied molecular orbital (HOMO) energy levels because p-type OSCs require suitable HOMO levels. Considering the carrier injection at electrodes and the stability in air, the molecules having HOMO levels between −5.4 and −5.0 eV were selected [26]. The HOMO levels of the extracted 220,000 molecules were calculated via the density functional theory (DFT) at the B3LYP/6-31G(d) level using Gaussian 16 [27]. Experimental geometries were used without structural optimization while considering the structures in solids and reducing the calculation time. We used a supercomputer system ITO at Kyushu University (dual central processing units (CPUs) of Intel Xeon Gold 6154 (Skylake-SP) with 192 GB memory per node). The secondary screening (Figure 1) indicated 28,681 compounds to fit within the range of the HOMO level.

TIs were calculated using the same supercomputer system, Gaussian 16, and a custom Python code *tcal* [28]. The TI between molecules A and B was calculated via DFT at the B3LYP/6-31G(d,p) level for monomers A and B and the dimer AB. The monomer molecular orbitals |A⟩ and |B⟩ of the monomers were calculated. The Fock matrix F and overlap matrix S were calculated for the dimer system. Finally, the TI $\left(t\right)$ was calculated using Equation (1) [11].

(1) $$\begin{array}{c} t=\frac{\left\langle A|F|B\right\rangle -\frac{1}{2}\left(\varepsilon_{A}+\varepsilon_{B}\right)\left\langle A|S|B\right\rangle }{1-{\left\langle A|S|B\right\rangle }^{2}}, \end{array}$$

where εA≡<A|F|A> and εB≡<B|F|B>. The molecular geometries were prepared as follows. The first molecule of each crystal structure data was selected as a central molecule. All pairs of the central and neighboring molecules were used for the calculation of TIs. The central molecule and each of neighboring molecules are assumed to be paired if the shortest distance between atoms separately belonging to them is less than the sum of van der Waals radii and 0.4 Å. This process was applied to 28,681 compounds selected after secondary screening. Gaussian jobs were executed using a maximum of 32 jobs with 9 CPU cores in parallel, and the total CPU time was approximately two months.

Apart from the intermolecular TI for general use, we developed an interatomic TI for further analysis [16]. The basis functions $\left|i\rangle \right.$ and $\left|j\rangle \right.$ of each atom were grouped, and the molecular orbitals were expressed as $\left|A\rangle \right.=\sum_{\alpha }^{A}\sum_{i}^{\alpha }a_{i}\left|i\rangle \right.$ and $\left|B\rangle \right.=\sum_{\beta }^{B}\sum_{j}^{\beta }b_{j}\left|j\rangle \right.$, where α and β are the indices of atoms, *i* and *j* are the indices of basis functions, and $a_{i}$ and $b_{j}$ are the coefficients of basis functions. Substituting this formula into the numerator of Equation (1) gives Equation (2).

(2) $$\begin{array}{c} t=\overset{A}{\underset{\alpha }{\sum }}\overset{B}{\underset{\beta }{\sum }}\overset{\alpha }{\underset{i}{\sum }}\overset{\beta }{\underset{j}{\sum }}a_{i}^{*}b_{j}\frac{\left\langle i|F|j\right\rangle -\frac{1}{2}\left(\varepsilon_{A}+\varepsilon_{B}\right)\left\langle i|S|j\right\rangle }{1-{\left\langle A|S|B\right\rangle }^{2}}=\overset{A}{\underset{\alpha }{\sum }}\overset{B}{\underset{\beta }{\sum }}u_{\alpha ,\beta }. \end{array}$$

Here, we define the interatomic TI $u_{\alpha ,\beta }$ as expressed in Equation (3).

(3) $$\begin{array}{c} u_{\alpha ,\beta }\equiv \overset{\alpha }{\underset{i}{\sum }}\overset{\beta }{\underset{j}{\sum }}a_{i}^{*}b_{j}\frac{\left\langle i|F|j\right\rangle -\frac{1}{2}\left(\varepsilon_{A}+\varepsilon_{B}\right)\left\langle i|S|j\right\rangle }{1-{\left\langle A|S|B\right\rangle }^{2}}. \end{array}$$

We used the denominator of Equation (1) as is without substitution to obtain the practical definition of $u_{\alpha ,\beta }$ because <A|S|B>2 is usually small with respect to 1. The sum of interatomic TIs is equal to the intermolecular TI. An example of the calculated interatomic TIs is shown in Figure S2. The interatomic TIs enable the analysis at the substructure level, which is common among various compounds.

## Results and discussion

**Figure 2.**
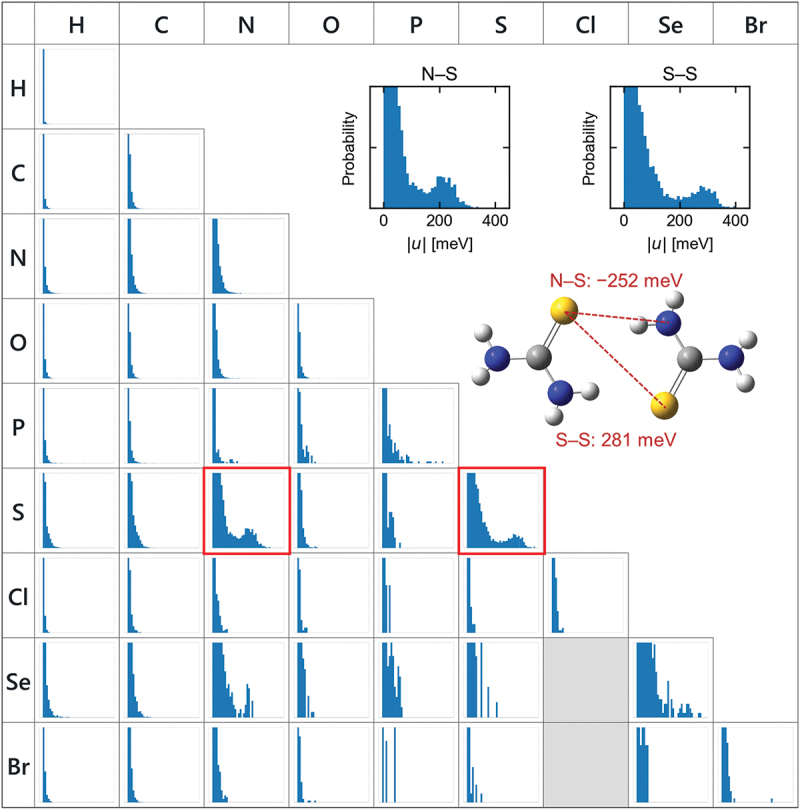
Histogram of interatomic transfer integrals (*u*) classified by element type.

The interatomic TIs of 27,718 compounds were calculated by excluding radicals and errors such as no convergence. Figure 2 shows the histograms of interatomic TIs for respective element pairs. The bottom axes of all histograms represent the absolute values of the interatomic TIs in the same range of 0–400 meV. The left axes of all histograms are the normalized probability in the same range; the integral of the histogram is always unity. The results indicate that interatomic TIs of nitrogen – sulfur and sulfur – sulfur pairs are high. Nitrogen – selenium and selenium – selenium pairs also show high interatomic TIs, while such data were fewer than those of nitrogen – sulfur and sulfur – sulfur pairs.

**Figure 3.**
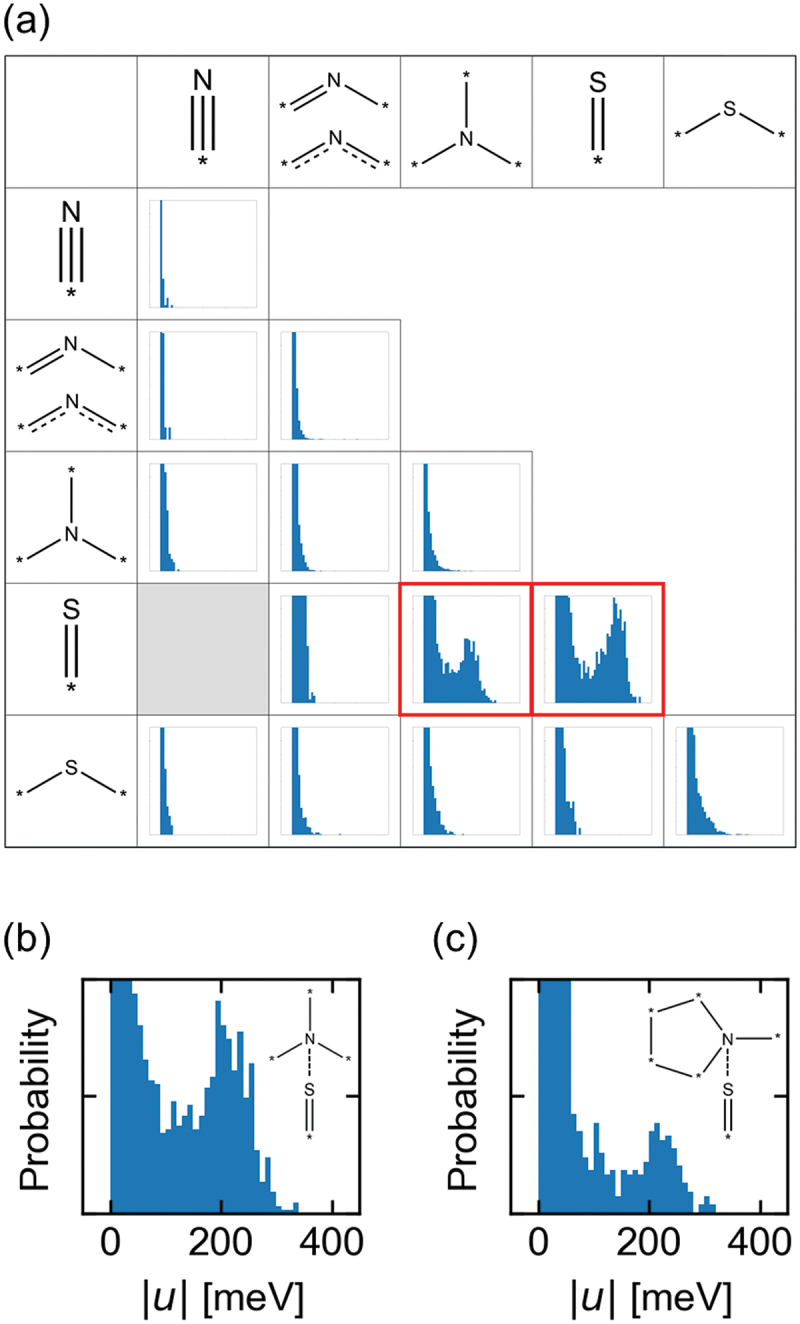
(a) Histogram of interatomic TIs (*u*) classified by the number of bonds and bond type. Histogram of interatomic TIs between (b) acyclic amine and thione and (c) cyclic amine and thione.

**Figure 4.**
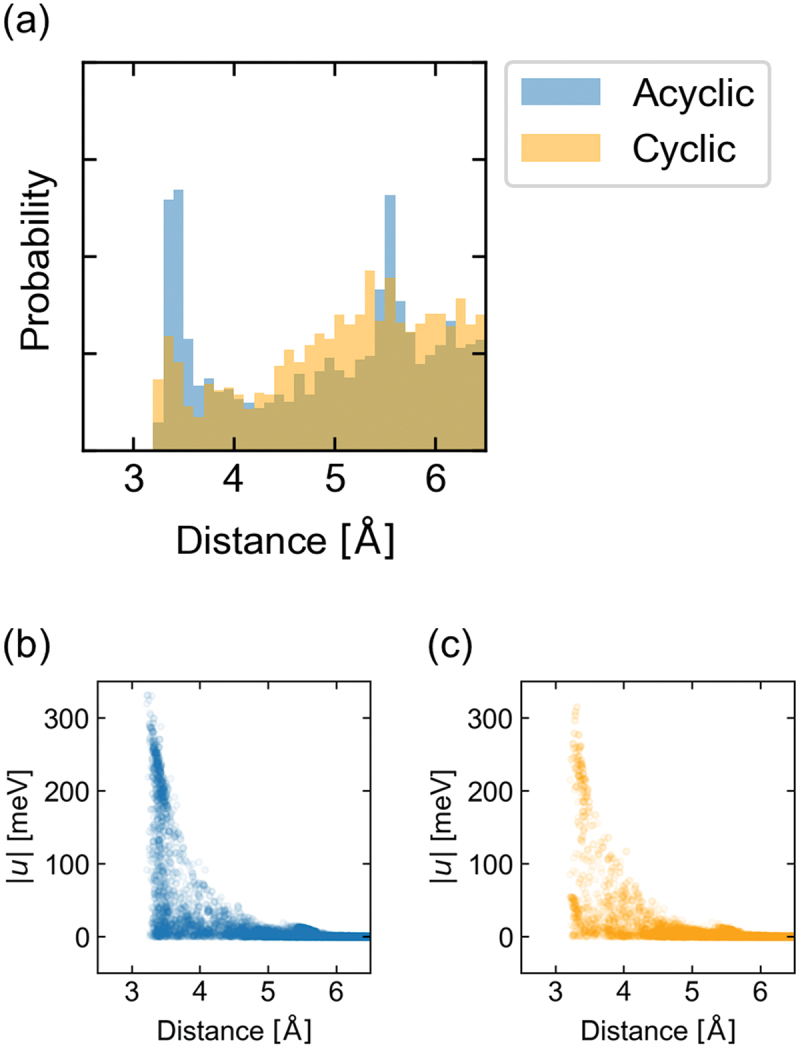
(a) Histogram of the distance between nitrogen and sulfur. Scatter plot of distances and interatomic TIs (*u*) of (b) acyclic amine and thione and (c) cyclic amine and thione.

Nitrogen and sulfur atoms were categorized into several atom types considering the number of bonds for more detailed analysis, as shown in Figure 3(a). The histograms indicate that interatomic TIs are high between (i) amine (NR_3_) and thione (S=R) and (ii) thione and thione. Amine can be further classified into cyclic and acyclic amines. Classification from the point of view Figures 3(b,c) indicates that TIs can be high in both cases; however, acyclic amines are better than cyclic amines. Figure 4a shows a histogram indicating the distance between nitrogen and sulfur atoms to investigate the high TIs of acyclic amine compounds. The pairs of acyclic nitrogen and sulfur atoms have a higher probability at shorter distances of 3.3–3.5 Å than the pairs of cyclic nitrogen and sulfur atoms. The value is close to the sum of the van der Waals radii of nitrogen and sulfur. One possible reason for the short distance of acyclic nitrogen compounds is the flexibility of the acyclic structure. These molecules often have hydrogen bonds to nitrogen. Hence, the flexibility facilitates the formation of hydrogen bonds and makes the distance short. Figures 4(b,c) show the correlation between interatomic distance and TIs for amine and thione. The clusters with high TIs of 200–250 meV at distances of 3.3–3.5 Å imply that a specific interaction between amine and thione exists. Schober et al. have reported related research, where they have performed DFT calculations of transfer integrals of 95,445 organic crystals [17]. A compound containing thione and amine groups was one of the candidates for organic semiconductors in their research.

**Figure 5.**
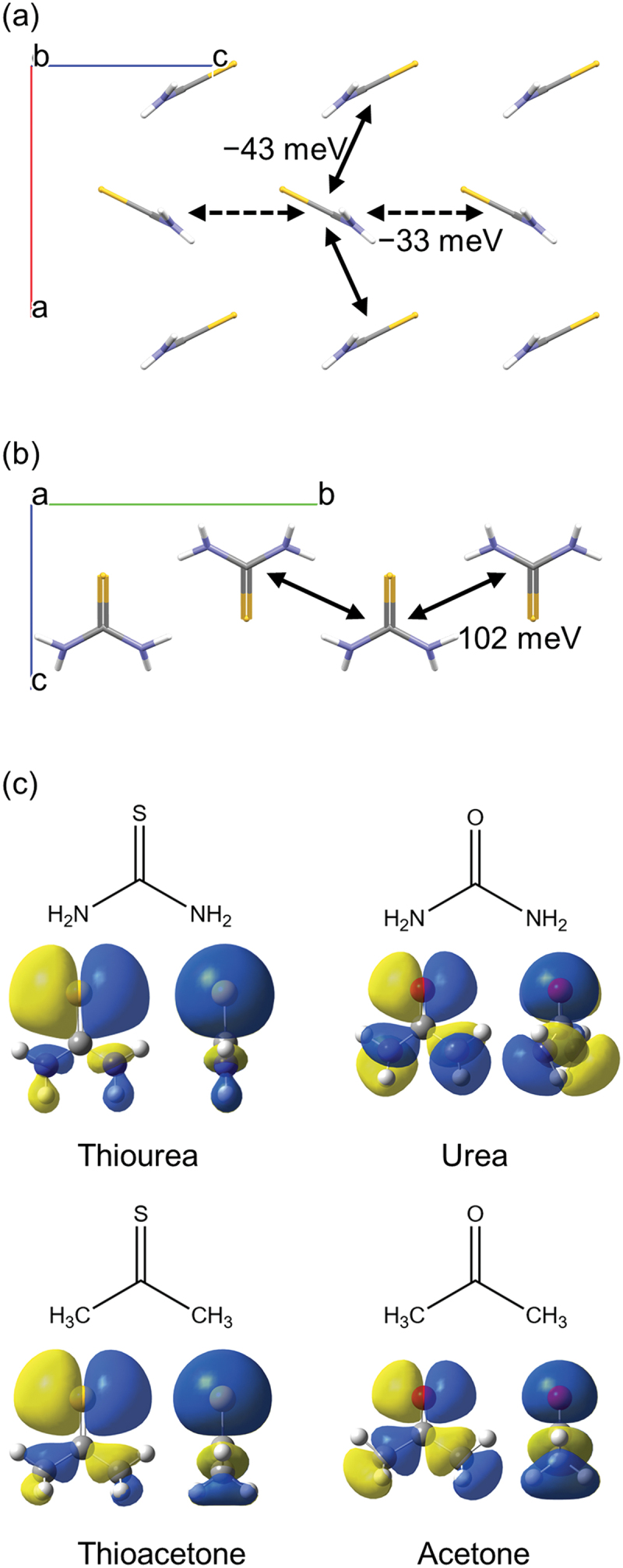
(a,b) Transfer integrals of thiourea. (c) The HOMO of thiourea, urea, thioacetone, and acetone.

Thiourea is the simplest compound with amine and thione. The charge carrier mobility of thiourea polycrystal was estimated as ~0.2 cm^2^ V^−1^ s^−1^ in 1970 via an uncommon method based on the Seebeck coefficient [23,24]. The mobility exhibited negative temperature dependence as expected in band conduction. The intermolecular TIs of thiourea crystal presented in Figures 5(a,b) show that many dimer pairs exhibit high TI values (102, −43, and −33 meV). The band calculation of the thiourea crystal at the PBEPBE/6-31 G(d,p) level shows small effective mass values of −1.4 $m_{0}$ and −1.6 $m_{0}$ in a two-dimensional plane at T point, where $m_{0}$ is the free electron mass (Figure S3). These values are comparable to those of high mobility OSCs [9]. In addition to thiourea, we found many other compounds having thiourea substructures exhibit high TIs (Figure S4), while the continuity of high TI network has not been fully investigated yet. Thus, thiourea and its derivatives can be unique OSCs in that they are non-aromatic and soluble in water.

Lastly, the HOMO (Figure 5c) and molecular orbital energy levels (Table 1) of thiourea, urea, thioacetone, and acetone were calculated to examine the cause of the high TIs of thiourea. The orbital of sulfur in thiourea is more expanded than that of oxygen in urea and contributes to high TIs between amine and thione, and thione and thione. The amine groups of thiourea increase the HOMO energy level by 0.42 eV compared to thioacetone and make it suitable for p-type semiconductors. The HOMO (−5.45 eV) and HOMO-1 (−5.68 eV) might be switched when incorporated into a crystal because both the energy levels are close, as shown in Table 1.

**Table 1.** Energy levels of thiourea, urea, thioacetone, and acetone (B3LYP/6-31 G(d,p)).

	HOMO−1 [eV]	HOMO [eV]	LUMO [eV]	LUMO+1 [eV]
Thiourea	−5.68	−5.45	0.24	1.21
Urea	−7.40	−6.79	1.43	1.73
Thioacetone	−7.70	−5.87	−1.91	2.22
Acetone	−9.43	−6.64	−0.30	2.40

## Conclusions

In summary, we devised interatomic TIs and systematically calculated the TIs for 27,718 compounds. The results indicated that the TIs of nitrogen – sulfur and sulfur – sulfur pairs were high. The TIs of thione (S=R) and acyclic amine (NR_3_) groups were preferable. The expanded molecular orbitals of thione and the short distance between sulfur and acyclic nitrogen increased TI values. Thiourea is a representative substructure containing thione and acyclic amine with high TIs. Several dimers with high TIs were obtained from the thiourea calculation. We consider that molecules with thiourea substructures have the potential for non-aromatic and water-soluble p-type OSCs.

## Supplementary Material

Supplemental Material

## References

[1] Sirringhaus H. 25th anniversary article: organic field-effect transistors: the path beyond amorphous silicon. Adv Mater. 2014 Mar;26(9):1319–6. doi: 10.1002/adma.201304346 24443057 PMC4515091

[2] Matsui H, Takeda Y, Tokito S. Flexible and printed organic transistors: from materials to integrated circuits. Organic Electron. 2019 Dec;75:105432. doi: 10.1016/j.orgel.2019.105432

[3] Yamamura A, Matsui H, Uno M, et al. Painting integrated complementary logic circuits for single-crystal organic transistors: a demonstration of a digital wireless communication sensing tag. Adv Electron Mater. 2017 Jul;3(7):1600456. doi: 10.1002/aelm.201600456

[4] Paterson AF, Singh S, Fallon KJ, et al. Recent progress in high-mobility organic transistors: a reality check. Adv Mater. 2018 Sep 6;30(36). doi: 10.1002/adma.201801079 30022536

[5] Fratini S, Nikolka M, Salleo A, et al. Charge transport in high-mobility conjugated polymers and molecular semiconductors. Nature Mater. 2020 May;19(5):491–502. doi: 10.1038/s41563-020-0647-2 32296138

[6] Sundar VC, Zaumseil J, Podzorov V, et al. Elastomeric transistor stamps: reversible probing of charge transport in organic crystals. Science. 2004 Mar 12;303(5664):1644–1646. doi: 10.1126/science.1094196 15016993

[7] Iino H, Usui T, Hanna J. Liquid crystals for organic thin-film transistors. Nat Commun. 2015 Apr 6;6(1):6828. doi: 10.1038/ncomms7828 PMC4403349 25857435

[8] Mitsui C, Okamoto T, Yamagishi M, et al. High-performance solution-processable N-shaped organic semiconducting materials with stabilized crystal phase. Adv Mater. 2014 Jul 9;26(26):4546. doi: 10.1002/adma.201400289 24811889

[9] Higashino T, Inoue S, Arai S, et al. Architecting layered crystalline organic semiconductors based on unsymmetric π-extended thienoacenes. Chem Mater. 2021 Sep 28;33(18):7379–7385. doi: 10.1021/acs.chemmater.1c01972

[10] Takimiya K, Bulgarevich K, Abbas M, et al. “Manipulation” of crystal structure by methylthiolation enabling ultrahigh mobility in a pyrene-based molecular semiconductor. Adv Mater. 2021 Aug;33(32):2102914. doi: 10.1002/adma.202102914 PMC11468581 34219291

[11] Coropceanu V, Cornil J, da Silva DA, et al. Charge transport in organic semiconductors. Chem Rev. 2007 Apr;107(4):926–952. doi: 10.1021/cr050140x 17378615

[12] Deng WQ, Goddard WA. Predictions of hole mobilities in oligoacene organic semiconductors from quantum mechanical calculations. J Phys Chem B. 2004 Jun 24;108(25):8614–8621. doi: 10.1021/jp0495848

[13] Cheng YC, Silbey RJ, da Silva DA, et al. Three-dimensional band structure and bandlike mobility in oligoacene single crystals: a theoretical investigation. J Chem Phys. 2003 Feb 22;118(8):3764–3774. doi: 10.1063/1.1539090

[14] Inoue S, Minemawari H, Tsutsumi J, et al. Effects of substituted alkyl chain length on solution-processable layered organic semiconductor crystals. Chem Mater. 2015 Jun 9;27(11):3809–3812. doi: 10.1021/acs.chemmater.5b00810

[15] Sokolov AN, Atahan-Evrenk S, Mondal R, et al. From computational discovery to experimental characterization of a high hole mobility organic crystal. Nat Commun. 2011 Aug 2;2(1):437. doi: 10.1038/ncomms1451 PMC3366639 21847111

[16] Inoue S, Higashino T, Arai S, et al. Regioisomeric control of layered crystallinity in solution-processable organic semiconductors. Chem Sci. 2020 Dec 14;11(46):12493–12505. doi: 10.1039/d0sc04461j 34976335 PMC8647348

[17] Schober C, Reuter K, Oberhofer H. Virtual screening for high carrier mobility in organic semiconductors. J Phys Chem Lett. 2016 Oct 6;7(19):3973–3977. doi: 10.1021/acs.jpclett.6b01657 27661442

[18] Kunkel C, Schober C, Margraf JT, et al. Finding the right bricks for molecular Legos: a data mining approach to organic semiconductor design. Chem Mater. 2019 Feb 12;31(3):969–978. doi: 10.1021/acs.chemmater.8b04436

[19] Matsuzawa NN, Arai H, Sasago M, et al. Massive theoretical screen of hole conducting organic materials in the heteroacene family by using a cloud-computing environment. J Phys Chem A. 2020 Mar 12;124(10):1981–1992. doi: 10.1021/acs.jpca.9b10998 32069044

[20] Kunkel C, Margraf JT, Chen K, et al. Active discovery of organic semiconductors. Nat Commun. 2021 Apr 23;12(1):2422. doi: 10.1038/s41467-021-22611-4 PMC8065160 33893287

[21] Homma Y, Ogawa H, Matsui H. Exploring organic cathode materials for lithium-ion batteries through fragment bonding and discharge simulation. J Phys Chem C. 2024 Feb 1;128(6):2304–2310. doi: 10.1021/acs.jpcc.3c06045

[22] Groom CR, Bruno IJ, Lightfoot MP, et al. The Cambridge structural database. Acta Crystallogr B Struct Sci Cryst Eng Mater. 2016 Apr;72(2):171–179. doi: 10.1107/S2052520616003954 PMC4822653 27048719

[23] Yoganarasimhan S, Sood R. Semiconductivity of thiourea. Philos Mag. 1970;22(179):1075–1080. doi: 10.1080/14786437008221078

[24] Shaaban SM, Elsayed BA, Shabana AA, et al. Temperature-dependence of the electrical-conductivity of urea and thiourea. Materials Letters. 1994 Nov;21(3–4):255–258. doi: 10.1016/0167-577x(94)90185-6

[25] Bruno IJ, Cole JC, Edgington PR, et al. New software for searching the Cambridge structural database and visualizing crystal structures. Acta Crystallogr B-Struct Sci Crystal Eng Mater. 2002 Jun;58(3):389–397. doi: 10.1107/S0108768102003324 12037360

[26] Zhao Y, Guo YL, Liu YQ. 25th anniversary article: recent advances in n-Type and ambipolar organic field-effect transistors. Adv Mater. 2013 Oct 11;25(38):5372–5391. doi: 10.1002/adma.201302315 24038388

[27] Frisch MJ, Trucks GW, Schlegel HB, et al. Gaussian 16, revision C.01. Wallingford (CT): Gaussian, Inc; 2016.

[28] Matsui H, Ozawa K. tcal: program for the calculation of transfer integral: GitHub repository. 2023. Available from: https://github.com/matsui-lab-yamagata/tcal

