# Supplementary material for: Statistical analysis of interatomic transfer integrals for exploring high-mobility organic semiconductors
Source: Sci Technol Adv Mater. 2024 May 17;25(1):2354652. doi: 10.1080/14686996.2024.2354652 (PMC11168228; doi:10.1080/14686996.2024.2354652)
Supplement: Supplemental Material [file TSTA_A_2354652_SM2726.docx]

# Supplementary material

Statistical Analysis of Interatomic Transfer Integrals for Exploring High-Mobility Organic Semiconductors

Koki Ozawa^a^, Tomoharu Okada^a^ and Hiroyuki Matsui^a^*

*^a^Research Center for Organic Electronics (ROEL), Yamagata University, Yonezawa, Japan*

# *[h-matsui@yz.yamagata-u.ac.jp](mailto:h-matsui@yz.yamagata-u.ac.jp)

Table S1. Comparison of HOMO energies by experiments (cyclic voltammetry) and calculations in different methods. Chemical structures of the seven compounds are shown in Figure S1.

| Refcode | BOSBIW | BOWLOP | ZIGPIR | ZODBUR | ZOGFIM | ZONQIE | ZUVRUF |
| --- | --- | --- | --- | --- | --- | --- | --- |
| B3LYP/6-31G(d) | −5.22 | −5.27 | −5.17 | −5.23 | −5.06 | −5.27 | −5.11 |
| B3LYP/6-311G(d) | −5.45 | −5.47 | −5.40 | −5.46 | −5.28 | −5.50 | −5.33 |
| B3LYP/6-31G+(d) | −5.55 | −5.50 | −5.46 | −5.52 | −5.34 | −5.55 | −5.38 |
| B3LYP/6-31G(d,p) | −5.24 | −5.27 | −5.17 | −5.24 | −5.07 | −5.28 | −5.12 |
| PBEPBE/6-31G(d,p) | −4.63 | −4.62 | −4.57 | −4.59 | −4.43 | −4.72 | −4.45 |
| HF/6-31G(d) | −7.19 | −7.16 | −7.17 | −7.00 | −7.06 | −6.92 | −7.23 |
| CCSD(T) | −5.10 | −5.62 | −5.35 | −5.49 | −5.66 | −5.59 | −5.65 |
| Experiment | −5.56^[1]^ | −5.51^[2]^ | −5.35^[3]^ | –5.51^[4]^ | −5.39^[5]^ | −5.48^[6]^ | –5.57^[7]^ |

| 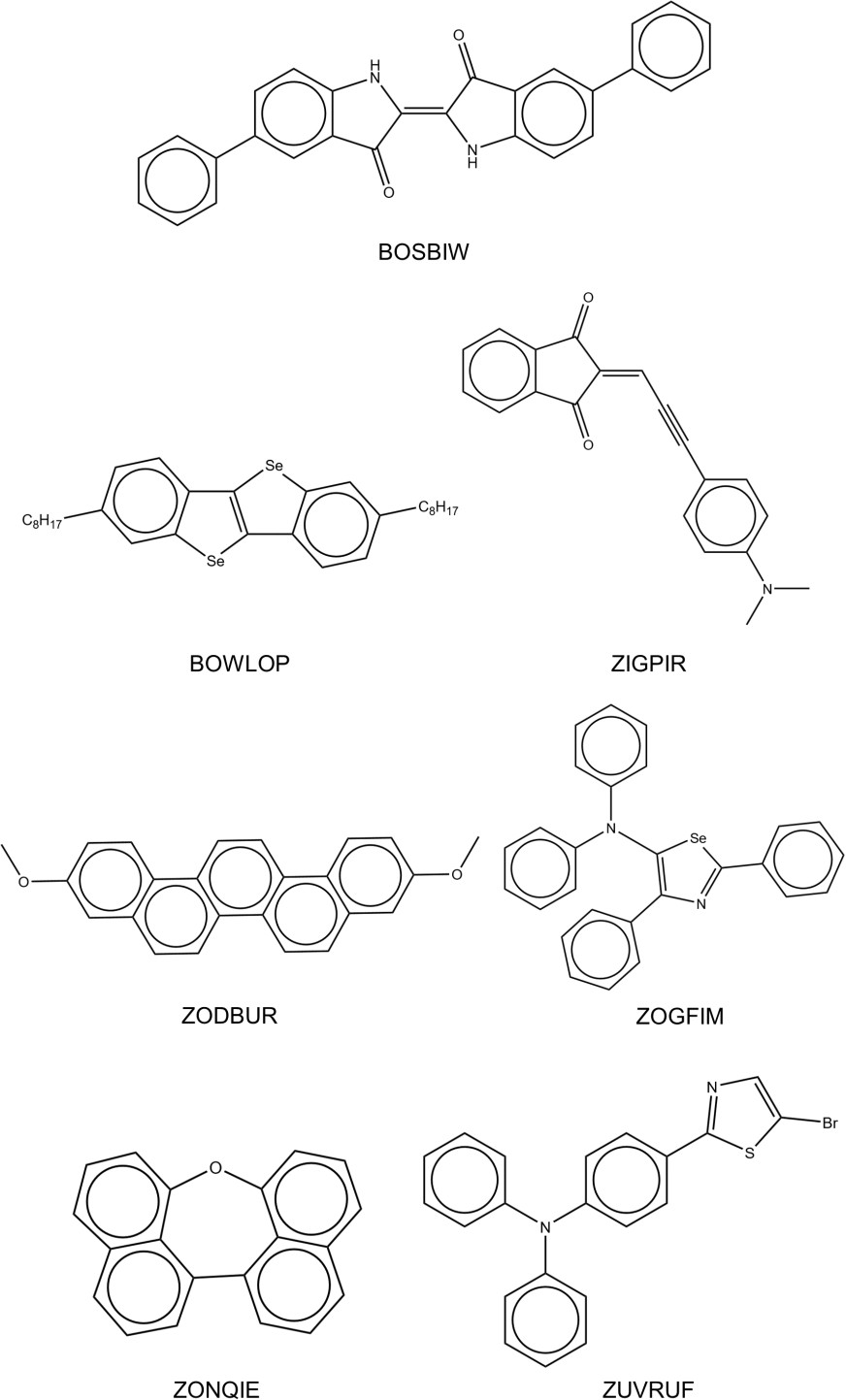 |
| --- |
| Figure S1. Compounds calculated in Table S1. |

| 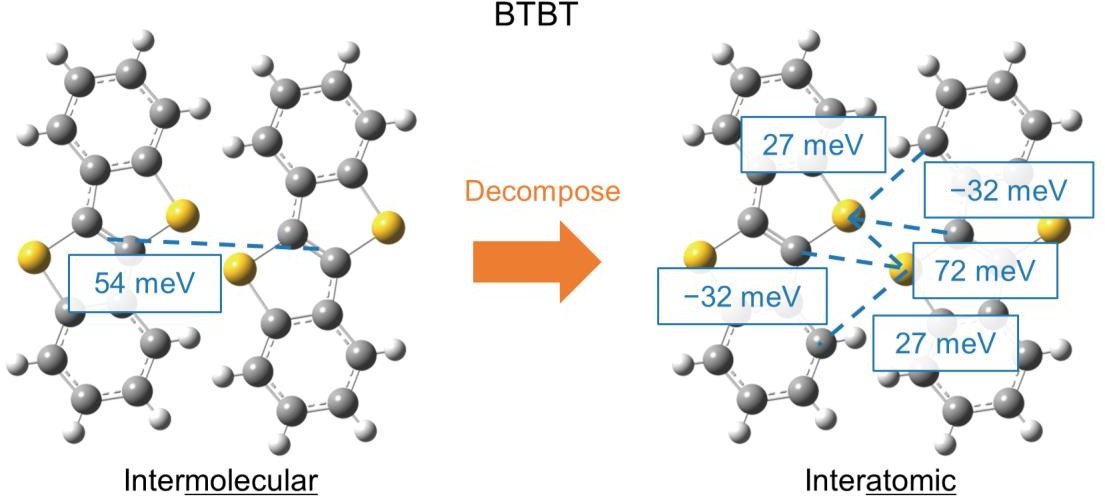 |
| --- |
| Figure S2. Explanation of intermolecular and interatomic transfer integrals. When the intermolecular transfer integral is decomposed, the interatomic transfer integral between sulfur is 72 meV. Gray atoms are carbon, yellow atoms are sulfur, and white atoms are hydrogen. |

| 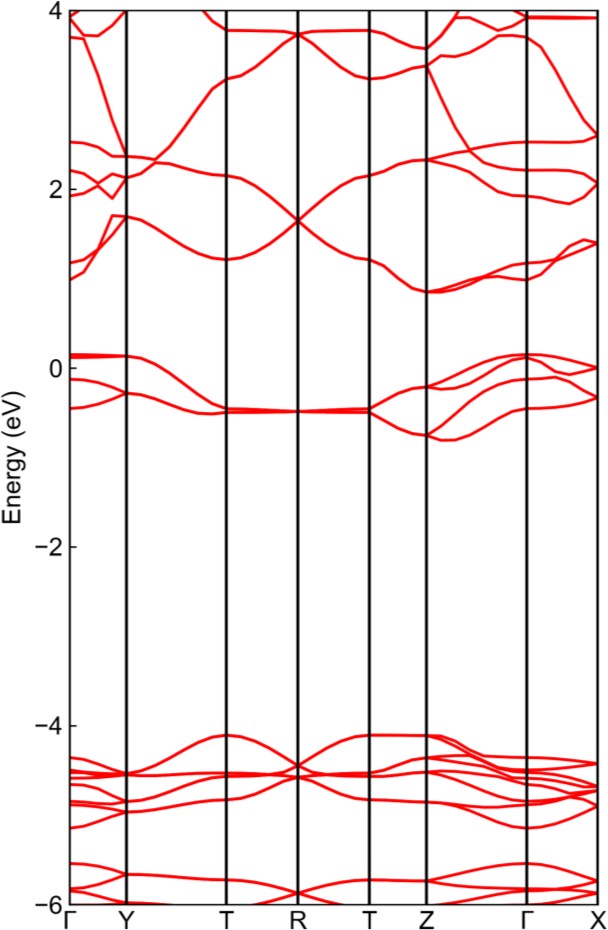 |
| --- |
| Figure S3. Band structure of thiourea. PBEBPE functional and 6-31G(d,p) basis set were used. Lattice parameter is *a* = 7.655(7) Å, *b* = 8.537(7) Å, *c* = 5.520(7) Å, *α* = 90°, *β* = 90°, and *γ* = 90°.  T: (*ka*, *kb*, *kc*) = (0, π/*b*, π/*c*) |

| 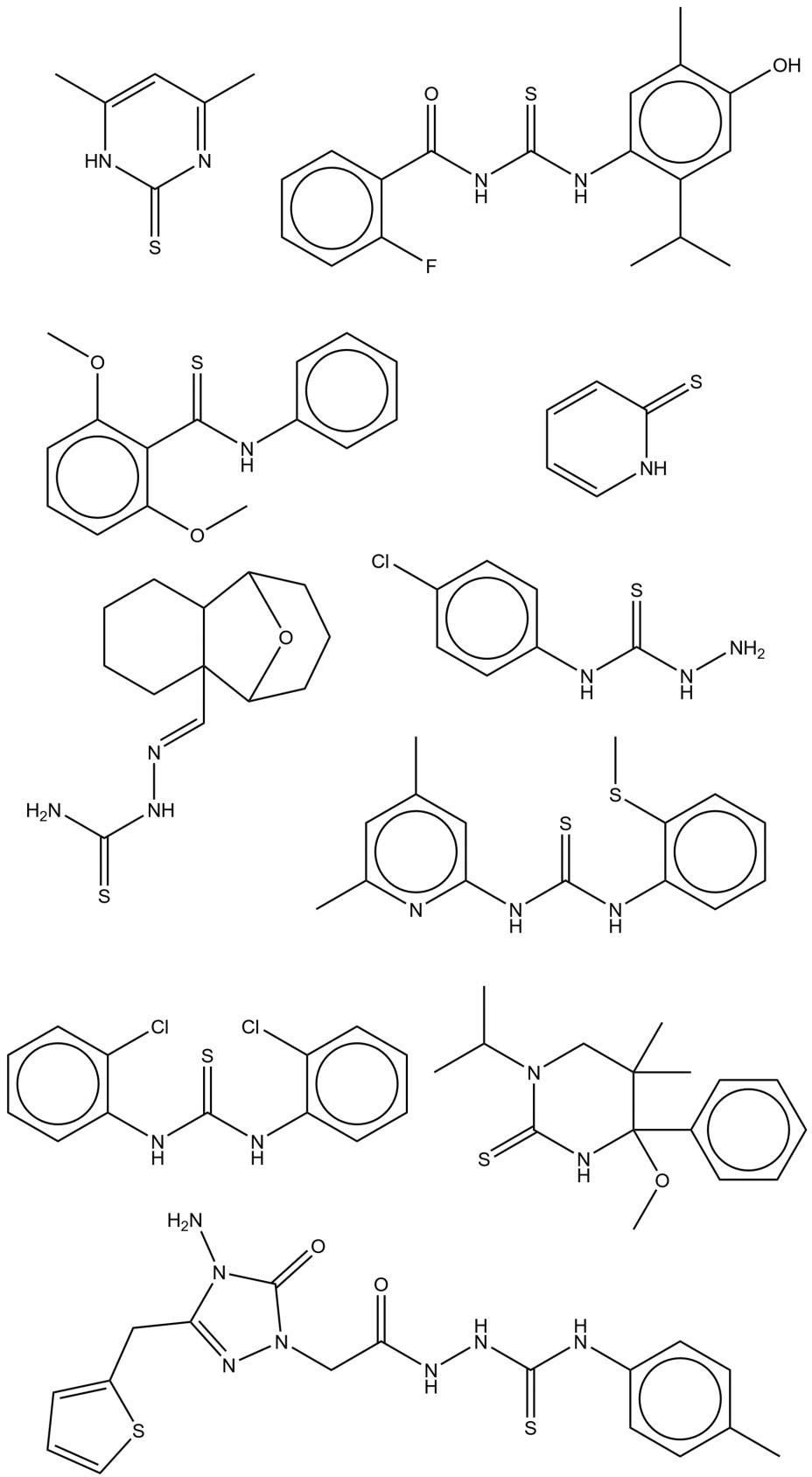 |
| --- |
| Figure S4. Example of molecules with high transfer integrals between nitrogen and sulfur. |

1. Pitayatanakul O, Higashino T, Kadoya T, et al. High performance ambipolar organic field-effect transistors based on indigo derivatives. Journal of Materials Chemistry C. 2014 Sep 17;43(2): 9311-9317. doi: 10.1039/c4tc01563k.
2. Izawa T, Miyazaki E, Takimiya K, Solution-Processible Organic Semiconductors Based on Selenophene-Containing Heteroarenes, 2,7-Dialkyl[1]benzoselenopheno[3,2-b][1]benzoselenop

-henes (Cn-BSBSs): Syntheses, Properties, Molecular Arrangements, and Field-Effect Transistor Characteristics. Chemistry of Materials. 2009 Mar 10;21(5) 903–912. doi: 10.1021/cm8030126.

1. Solanke P, Růžička A, Mikysek T, et al. From Linear to T-Shaped Indan-1,3-dione Push–Pull Molecules: A Comparative Study. HELVETICA chimica acta. 2018 Aug 16;101(8) e201800090. doi: 10.1002/hlca.201800090.
2. Mori H, Chen X, Chang N, et al. Synthesis of Methoxy-Substituted Picenes: Substitution Position Effect on Their Electronic and Single-Crystal Structures. The Journal of Organic Chemistry. 2014 Jun 6;79(11) 4973–4983. doi: 10.1021/jo500543h.
3. Murai T, Yamaguchi K, Hori F, et al. Reaction of Selenoamide Dianions with Thio- and Selenoformamides Leading to the Formation of 5-Aminoselenazoles: Photophysical and Electrochemical Properties. The Journal of Organic Chemistry. 2014 Apr 28;79(11) 4930-4939. doi: 10.1021/jo500499g.
4. Dobelmann L, Parham A, Büsing A, et al. First synthesis of naphthalene annulated oxepins. RSC Advances. 2014 Nov 6;4(105) 60473–60477. doi: 10.1039/C4RA10652K.
5. Tao T, Ma B, Peng Y, et al. Asymmetrical/Symmetrical D−π–A/D−π–D Thiazole-Containing

Aromatic Heterocyclic Fluorescent Compounds Having the Same Triphenylamino Chromophores. The Journal of Organic Chemistry. 2013 Aug 13;78(17) 8669-8679. doi: 10.1021/jo401384g.
